# On the quantification of the dosimetric accuracy of collapsed cone convolution superposition (CCCS) algorithm for small lung volumes using IMRT

**DOI:** 10.1120/jacmp.v13i3.3751

**Published:** 2012-05-10

**Authors:** Oscar I. Calvo, Alonso N. Gutiérrez, Sotirios Stathakis, Carlos Esquivel, Nikos Papanikolaou

**Affiliations:** ^1^ Department of Radiation Oncology, School of Medicine Cancer Therapy & Research Center at The University of Texas Health Science Center San Antonio San Antonio TX 78229 USA

**Keywords:** Monte Carlo, CCCS, small fields, SBRT

## Abstract

Specialized techniques that make use of small field dosimetry are common practice in today's clinics. These new techniques represent a big challenge to the treatment planning systems due to the lack of lateral electronic equilibrium. Because of this, the necessity of planning systems to overcome such difficulties and provide an accurate representation of the true value is of significant importance. ${\text{Pinnacle}}^{3}$ is one such planning system. During the IMRT optimization process, ${\text{Pinnacle}}^{3}$ treatment planning system allows the user to specify a minimum segment size which results in multiple beams composed of several subsets of different widths. In this study, the accuracy of the engine dose calculation, collapsed cone convolution superposition algorithm (CCCS) used by ${\text{Pinnacle}}^{3}$, was quantified by Monte Carlo simulations, ionization chamber, and Kodak extended dose range film (EDR2) measurements for 11 SBRT lung patients. Lesions were < 3.0 cm in maximal diameter and $<27.0\;{\text{cm}}^{3}$ in volume. The Monte Carlo EGSnrc\BEAMnrc and EGS4\MCSIM were used in the comparison. The minimum segment size allowable during optimization had a direct impact on the number of monitor units calculated for each beam. Plans with the smallest minimum segment size ($0.1\;{\text{cm}}^{2}$ to $2.0\;{\text{cm}}^{2}$) had the largest number of MUs. Although PTV coverage remained unaffected, the segment size did have an effect on the dose to the organs at risk. ${\text{Pinnacle}}^{3}$‐calculated PTV mean doses were in agreement with Monte Carlo‐calculated mean doses to within 5.6% for all plans. On average, the mean dose difference between Monte Carlo and ${\text{Pinnacle}}^{3}$ for all 88 plans was 1.38%. The largest discrepancy in maximum dose was 5.8%, and was noted for one of the plans using a minimum segment size of $0.1\;{\text{cm}}^{2}$. For minimum dose to the PTV, a maximum discrepancy between Monte Carlo and ${\text{Pinnacle}}^{3}$ was noted of 12.5% for a plan using a $6.0\;{\text{cm}}^{2}$ minimum segment size. Agreement between point dose measurements and ${\text{Pinnacle}}^{3}$‐calculated doses were on average within 0.7% in both phantoms. The profiles show a good agreement between ${\text{Pinnacle}}^{3}$, Monte Carlo, and EDR2 film. The gamma index and the isodose lines support the result.

PACS number: 87.56.bd

## I. INTRODUCTION

The growing use of more specialized techniques, such as intracranial and extra‐cranial stereotactic radiotherapy (SRT) in which small fields with electronic disequilibrium are present, has been subject to investigation by many researchers.^(^ ^1^ ^)^ Intensity‐modulated radiotherapy (IMRT) plans make use of several superimposed small fields to achieve a highly conformal dose distribution to the target volume and spare healthy tissue.

IMRT treatment planning systems calculate an ideal intensity map for each field using optimization routines with physical dose objectives set by the user.^(^ ^2^ ^)^ Once the optimization completes, the optimized fluence for each field of the plan is realized into several small segments. These segments of small aperture are generated by the segmentation process that produces the delivery sequence of the multileaf collimator. Under such conditions of small field geometries, the electronic equilibrium can be lost, making it challenging for the dose calculation algorithm to accurately predict the dose, especially in the presence of tissue heterogeneities.^(^ ^1^ ^)^

IMRT planning utilizes iterative optimization techniques, during which the dose needs to be continuously calculated. To improve the efficiency and speed of such implementations, a pencil beam algorithm is typically used for the intermediate (iterative) dose calculation steps, thus introducing convergence errors.^(^ ^3^ ^)^ It is important to always perform a full 3D dose calculation at the conclusion of the optimization to evaluate the dose the patient will truly receive, especially since it is known that the pencil beam implementations are unreliable in the presence of tissue heterogeneities.

The traditional two‐step process of IMRT inverse planning may produce more segments and MUs than necessary—largely due to the conversion of the fluence profiles to deliverable MLC settings. However, with more advance techniques like direct machine parameter optimization (DMPO), the plan will not degrade because of the lack of post processing (no need of fluence profiles conversion to MLC settings).^(^ ^4^ ^)^

When calculating the dose in a low‐density medium such as lung, the use of narrow beams may produce significant perturbations that are energy and density dependent and ultimately affect the accuracy of the dose calculation. This problem is more pronounced when the TPS uses simple, one‐dimensional density scaling.^(^ ^5^ ^–^ ^8^ ^)^ The level of accuracy improves with the use of sophisticated treatment planning algorithms^(^ ^9^ ^–^ ^17^ ^)^ where multisource modeling is included, allowing a more accurate dose prediction for small fields and under non‐equilibrium conditions. ^(^ ^18^ ^,^ ^19^ ^)^ It has been shown that the accuracy of small field dosimetry is greatly improved when Monte Carlo simulations are employed, especially for beam sizes less than 3.0 by $3.0\;{\text{cm}}^{2}$ in inhomogeneous media.^(^ ^20^ ^–^ ^23^ ^)^

Currently, the model‐based dose calculation methods considered most accurate in radiotherapy are Monte Carlo transport and the convolution/superposition method.^(^ ^24^ ^)^ Researchers have reported various techniques and devices used for obtaining dosimetric input data for small and complicated photon beams.^(^ ^25^ ^–^ ^29^ ^)^ Francescon et al.^(^ ^30^ ^)^ reported that ${\text{Pinnacle}}^{3}$ overestimates the dose by up to 8% for narrow 1.0 cm wide photon segments. Azcona et al.^(^ ^31^ ^)^ reported an overestimation of calculated dose between 4% and 14% for fields of widths between 1 and 3 cm.

Sophisticated Monte Carlo codes are becoming available in parallel to the emerging use of small field radiotherapy techniques.^(^ ^32^ ^)^ By explicitly modeling the particle transport, complete Monte Carlo simulations are expected to result in the highest dose calculation accuracy^(^ ^33^ ^)^ and would become the standard for future planning systems. However, due to the long time required for a MC simulation, clinical implementation is currently not possible.

The quantification of the dose calculation accuracy for treatment planning systems is a matter of high importance in radiotherapy, as inaccuracies may lead to patient complications. This study has a two‐fold purpose: first, the dose calculated using the collapsed cone convolution superposition method used in ${\text{Pinnacle}}^{3}$ TPS is compared against Monte Carlo dose calculations, ion chamber, and EDR2 film measurements; second, the effect of the smallest segment size allowed during optimization is examined in terms of dose calculation accuracy, and dose to organs at risk and PTV coverage. Although several studies exist that directly compare the CCCS dose calculations against Monte Carlo calculations, there has not been a study to date that includes the above stated purposes.

## II. MATERIALS AND METHODS

### A.1 Patient selection

Eleven patients (n=11) were randomly chosen for this study. All the patients were treated using a stereotactic body radiation therapy (SBRT) technique. The patient selections were restricted such that the tumor size was < 3.0 cm in maximum diameter and the volume was < 27.0 cc. The location and target volumes of each patient are shown in Table 1.

**Table 1:** PTV location and volume size for all patients.

	*Location*	*Volume Size*
Patient 1	Mid right	$1.4\;{\text{cm}}^{3}$
Patient 2	Lower right	$5.0\;{\text{cm}}^{3}$
Patient 3	Upper left	$11.9\;{\text{cm}}^{3}$
Patient 4	Mid right	$19.1\;{\text{cm}}^{3}$
Patient 5	Lower right	$13.6\;{\text{cm}}^{3}$
Patient 6	Mid right	$7.7\;{\text{cm}}^{3}$
Patient 7	Upper right	$8.0\;{\text{cm}}^{3}$
Patient 8	Upper left	$22.4\;{\text{cm}}^{3}$
Patient 9	Mid upper‐right	$17.0\;{\text{cm}}^{3}$
Patient 10	Lower left	$10.5\;{\text{cm}}^{3}$
Patient 11	Lower left	$9.1\;{\text{cm}}^{3}$

All patients were scanned in the supine position using a 16 slice GE LightSpeed (GE Medical, Waukesha WI), and immobilized using the Body Pro‐Lok system (CIVCO Medical Solutions, Iowa City IA). A single radiation oncologist delineated the gross tumor volume (GTV) and organs at risk (OAR) using a free breathing scan. CT images and structures were exported to ${\text{Pinnacle}}^{3}$ (Philips Medical, Fitchburg WI) treatment planning system through DICOM and DICOM‐RT.

### A.2 Patients' treatment plans

For each patient, eight optimized plans were created varying the minimum allowable segment size, while maintaining the same optimization dose constraints. The dose prescribed to the target was 45.0 Gy in three fractions (see Table 2). Five coplanar beams were used in all cases; these were optimally placed in order to avoid critical organs. All plans were optimized for delivery using a Varian 2100EX (Varian Medical Systems, Palo Alto, CA) 6 MV photon beam equipped with a 120 Millennium multileaf collimator (MLC). The minimum allowable segment sizes were varied from $0.1\;{\text{cm}}^{2}$ to $6.0\;{\text{cm}}^{2}$ (0.1, 0.25, 1.0, 2.0, 3.0, 4.0, 5.0, and $6.0\;{\text{cm}}^{2}$). Smaller segment sizes allow for higher spatial resolution of the optimized fluence map and, thus, permit greater flexibility during optimization. However, the use of smaller segment sizes could introduce unwanted electronic disequilibrium effects. On the other hand, by limiting the maximum size to $6.0\;{\text{cm}}^{2}$, the optimization is more restricted and might fail to provide an optimal solution. It should be noted that the minimum size of the segment also affects the number of segments created and total number of monitor units (MU) required for the delivery.

**Table 2:** Optimization parameters used for the PTV and OARs in ${\text{Pinnacle}}^{3}$ planning system.

*Organ*	*Volume (%)*	*Dose(Gy)*	*Type of Constraint*
Target	‐	45	Min Dose
Lung (Isp)	90	10	Max DVH
Lung (Isp)	50	20	Max DVH
Lung (Isp)	45	10	Max DVH
Lung (Cntrl)	90	1	Max DVH
Lung (Cntrl)	50	2.5	Max DVH
Lung (Cntrl)	10	5	Max Dose
Spinal Cord	10	10	Max DVH
Trachea	50	5	Max DVH
Trachea	10	10	Max DVH
Esophagus	10	5	Max DVH
Heart	10	5	Max Dose

The direct machine parameter optimization (DMPO) within ${\text{Pinnacle}}^{3}$ was used during optimization for all plans. Utilizing the DMPO option eliminates the need for optimization conversion and, beams' fluence generated correspond to deliverable ones.

After optimization, a final dose calculation using the collapsed cone convolution superposition (CCCS) algorithm^(^ ^9^ ^)^ was performed. Dose grid size used for calculations was 0.2 cm by 0.2 cm by 0.2 cm. Special care was taken during the definition of the dose grid extent in order to ensure complete enclosure of all OARs.

### A.3 Monte Carlo calculations

**Figure 1:**
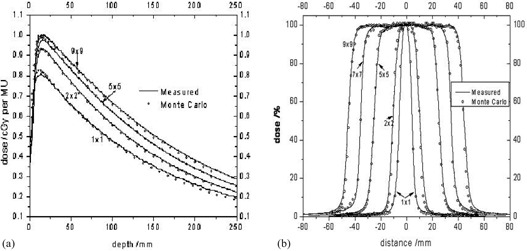
Monte Carlo vs. measurements for several field sizes: a) PDDs, and b) profiles.

Monte Carlo calculations for each of the optimized fluence plans were performed using both patient geometry and two phantom geometries. CT scans from each patient and the CT scans of the two phantoms were converted to Monte Carlo geometry. A total of 3 by 88 = 264 Monte Carlo dose calculations were performed. Monte Carlo calculations were performed in two steps. First, a phase space file of the linear accelerator was created using the EGSnrc/BEAMnrc system.^(^ ^34^ ^)^ Geometry and material composition used in the simulation were based on the specifications of the linear accelerator treatment head as provided by the manufacturer. The cutoff energies used for the simulations were 700 keV for electrons and 10 keV for photons. Energy thresholds for X‐ray production (AE) and for bremsstrahlung production (AP) were 700 keV and 10 keV, respectively. Results of the LINAC simulation were in agreement (±1% or within 1 mm) with the measured PDD curves and the dose profiles at various depths in water obtained with a 0.125 cc PTW‐Freiburg ion chamber. After the Monte Carlo commissioning of the linear accelerator, a phase space file was created before the jaws in order to be used in the dose calculation simulations (Fig. 1).

The Monte Carlo dose calculations were performed using the EGS4/MCSIM user code. The EGS4/MCSIM code system can accurately calculate patient dose distributions by simulating the accelerator head leakage, MLC leaf leakage and scatter, and the effect of beam modifiers such as collimator jaws, wedges, and blocks. Furthermore, calculation times are 10–30 times faster than other widely available general‐purpose Monte Carlo codes.^(^ ^35^ ^)^

The EGS4/MCSIM code was used in this work due to its extended functionality. The code is capable of calculating dose in a patient given the intensity map (ODM) or the Radiation Therapy Plan (RTP) file, which includes patient setup parameters and beam and leaf‐sequence information. The ODM files generated by ${\text{Pinnacle}}^{3}$ were used as input for EGS4/MCSIM. Based on the dose per incident particle, as derived from calculations using calibration conditions, EGS4/MCSIM can calculate the absolute dose to the patient. Moreover, if contours exist in the patient geometry from computed tomography (CT) images, doses to each organ may be calculated and DVHs generated for all contoured organs.

### A. 4 Ion chamber and film measurements

**Figure 2:**
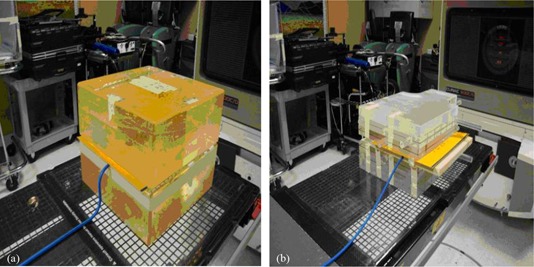
Setup geometries for film and ionization chamber measurements: (a) Plexiglass homogeneous phantom, and (b) StandardImaging heterogeneous phantom.

For each of the resulting 88 plans, two IMRT QA verification plans were created using a homogenous and heterogeneous phantom (Fig. 2). The heterogeneous phantom (Standard Imaging, Middleton WI) was used in order to mimic the patient geometry during radiation delivery. Each phantom was structured so that ionization chamber could be incorporated, as well as an extended dose radiographic (EDR2) film (Kodak Inc., Rochester NY). The film was placed within the phantom to measure the planar dose distribution. Verification plans were calculated and delivered with all of the beams positioned in planned angles. This resulted in 176 verification plans.

A vented cylindrical ionization chamber (PTW N31003, New York NY) with a sensitive volume of 0.3 cc was used for all measurements. Measured point doses from the ion chamber were compared against their respective calculated point dose from the TPS. The film dose measurements were compared to their respective calculated planar doses exported from ${\text{Pinnacle}}^{3}$. Film calibration was performed by exposing films to a dose range of 0.1 to 4.0 Gy using a step wedge with known dose values at the center of each step.^(^ ^36^ ^)^ All films were scanned on a Vidar 16‐bit scanner (VIDAR Systems Corporation Herndon, VA) and analyzed using the RIT113v5 (Radiological Imaging Technology, Colorado Springs, CO), software platform.

## III. RESULTS

### A.1 Effect of minimum segment size on plan quality

The minimum segment size allowable during optimization had a direct impact on the number of monitor units calculated for each beam. Plans with the smallest minimum segment size ($0.1\;{\text{cm}}^{2}$ to $2.0\;{\text{cm}}^{2}$) had the largest number of MUs per field (Table 3). Dose statistics for the planning target volumes of all of the patients are shown in Table 4. Small variations in the PTV dose statistics were noted, leading to the conclusion that there is no significant dependence of the PTV coverage on the minimum allowable segment size.

**Table 3:** Mean MU per field as a function of minimum segment size for each patient calculated using the CCCS algorithm.

				*Minimum Segment Field Size*			
	*0.1*	*0.25*	*1.0*	*2.0*	*3.0*	*4.0*	*5.0*	*6.0*
Patient 1	363	378	357	381	294	270	263	258
Patient 2	325	324	313	307	288	275	282	271
Patient 3	362	366	372	320	318	301	295	287
Patient 4	391	391	402	462	355	321	314	285
Patient 5	365	368	347	371	320	314	303	298
Patient 6	334	335	324	405	294	288	287	274
Patient 7	344	345	334	308	304	300	289	276
Patient 8	359	357	344	433	327	318	313	298
Patient 9	368	341	358	365	337	336	336	321
Patient 10	355	356	357	366	311	303	299	282
Patient 11	368	370	332	401	326	324	314	298
Mean	358	357	349	374	316	305	300	286

**Table 4:** ${\text{Pinnacle}}^{3}$ PTV dose statistics for all patients as a function of segment size.

*Segment Size*	*Minimum Dose (cGy)*	*Maximum Dose (cGy)*	*Mean Dose (cGy)*	*Dose STD (cGy)*
$0.1\;{\text{cm}}^{2}$	3978.8	4791.2	4544.7	115.4
$0.25\;{\text{cm}}^{2}$	3964.6	4619.1	4429.5	99.0
$1.0\;{\text{cm}}^{2}$	4019.1	4590.7	4467.4	90.0
$2.0\;{\text{cm}}^{2}$	4069.9	4656.0	4467.3	77.5
$3.0\;{\text{cm}}^{2}$	4016.1	4600.0	4438.3	81.6
$4.0\;{\text{cm}}^{2}$	4023.5	4603.0	4441.4	81.7
$5.0\;{\text{cm}}^{2}$	4025.7	4612.3	4443.6	83.5
$6.0\;{\text{cm}}^{2}$	4004.8	4623.0	4431.2	86.6

Although PTV coverage remained unaffected, the segment size did have an effect on the dose to the organs at risk. When segment sizes are large, the minimum segment shape is comparable to the aperture size and thereby limits the spatial resolution of the deliverable fluence. This limitation affects the sparing of critical structures, particularly those adjacent to the target volume. The dose to the organs at risk was shown to increase overall for very small and large minimum segment sizes allowable (Table 5).

**Table 5:** Mean (cGy) and maximum dose (cGy) to the lung and spinal cord for each patient plan calculated.

*#*	*Site*		$0.1\;cm^{2}$	$0.25\;cm^{2}$	$1\;cm^{2}$	$2\;cm^{2}$	$3\;cm^{2}$	$4\;cm^{2}$	$5\;cm^{2}$	$6\;cm^{2}$
1	Lung	Mean	349.0	344.3	348.7	350.2	356.7	346.9	348.3	351.4
		Max	4500.2	4482.7	4551.6	4613.3	4589.2	4579.8	4593	4629.2
	Spinal	Mean	589.2	613.8	612	672	539.7	706.3	717.2	586.6
	Cord	Max	787.8	852.5	721	920.1	620.7	836.9	851.6	661.4
2	Lung	Mean	494.3	499	506.8	520.3	493.2	502.4	501.4	501.5
		Max	4533.3	4803.1	4890.4	4639.7	4532.7	4595	4592.2	4603.8
	Spinal	Mean	74.6	86.7	88.7	70.2	70.9	72.2	71.6	73.7
	Cord	Max	237.5	290.6	320.3	185.5	226.6	241.1	218.1	225.9
3	Lung	Mean	420.3	481.3	503.8	511.9	504	504.9	508.8	515.5
		Max	5748.8	4643.9	4628.4	4601.8	4607.8	4591.9	4589.4	4598.6
	Spinal	Mean	17.2	62.9	52.2	43.6	45.04	42.8	39.4	39.8
	Cord	Max	32.4	128	87.9	87.1	125.7	81.5	73	75.5
4	Lung	Mean	516	516.2	522.1	537.8	524.9	526.4	526.7	521.3
		Max	4636.2	4636. 8	4672.2	4844.5	4678.9	4680.7	4699.4	4729.6
	Spinal	Mean	680.9	684.6	684.7	612.6	686	686.6	679.3	676.2
	Cord	Max	1285.3	1287.7	1302.7	1556.9	1300.7	1274.4	1278	1257.6
5	Lung	Mean	262.3	265.8	265.8	266.2	270.9	267.7	266.7	268.8
		Max	4495.8	4494.6	4494.6	4541.8	4536.9	4540.7	4557.7	4570.2
	Spinal	Mean	7.3	7.4	7.4	8.6	8.2	8	7.7	8
	Cord	Max	15.7	15.7	15.7	17.3	163	15.8	16.1	16.5
6	Lung	Mean	334.5	337.8	335.1	323.5	335.3	338	338.4	342.1
		Max	4555.8	4610.2	4568.7	4606.3	4550.7	4556.1	4561.6	4552.2
	Spinal	Mean	286	286.8	316.2	182.4	281.6	285.2	283.5	314.1
	Cord	Max	763.9	676.7	767.4	697.3	742.8	729	722.2	745.4
7	Lung	Mean	293.2	294.7	296.4	301.9	299.7	298.6	299.2	299.5
		Max	4573.8	4591.4	4617.3	4625.4	4592.7	4601.3	4610	4576.1
	Spinal	Mean	66.4	66.1	66.8	65.8	61.5	60.8	59	57.2
	Cord	Max	154.1	153.2	154.4	147.4	141	139.2	131.8	127.7
8	Lung	Mean	229.5	229	231.2	234.1	231.8	230.9	231	232.7
		Max	4624.3	4598.5	4603.3	4709.6	4615.1	4618.6	4637	4626.8
	Spinal	Mean	68.1	69	69.9	57.9	69.3	68.4	67.6	75.3
	Cord	Max	405.4	405.7	417.4	373.3	415.4	403.9	395.3	425.3
9	Lung	Mean	584.2	571	584.2	606.1	583.7	586.1	586.4	590.6
		Max	1587.1	4464.6	4569.2	4609.1	4544.4	4554.8	4559.2	4554.4
	Spinal	Mean	214.3	317.4	216.5	174.8	212.8	213.2	212.8	224.5
	Cord	Max	461.3	583.8	468.7	422.9	467.1	457.3	453.9	440.4
10	Lung	Mean	314.8	314.7	315.6	321.9	313.8	314.5	314.9	313.7
		Max	4629.6	4626.1	4645.8	4719	4667.6	4659	4668.2	4636.2
	Spinal	Mean	642.5	641.4	633.7	598.1	639.7	635.3	634.8	653.9
	Cord	Max	1163.4	1163.2	1137.3	1120.1	1139.1	1142.9	1144.3	1131.8
11	Lung	Mean	362.6	362.4	364	607.4	367.4	368.9	367.2	368.9
		Max	4555.9	4553.5	4548.5	4678.6	4568.2	4559	4566.5	4543
	Spinal	Mean	369.4	369.1	364	639.8	361.6	364	365	379.3
	Cord	Max	572.4	572.1	571.5	748.5	573	574	573.4	574

**Figure 3:**
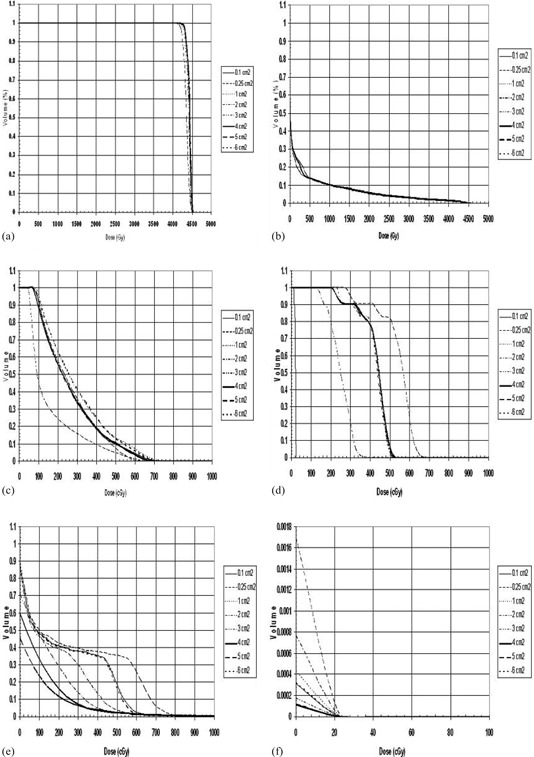
Dose volume histogram (DVH) of all eight plans for patient 1 for: (a) the PTV, (b) the lung, (c) spinal cord, (d) the esophagus, (e) the trachea, and (f) the heart.

Small differences were noted in the DVHs as the minimum segment sizes were varied for the target volumes (Fig. 3). Larger discrepancies were witnessed in the DVHs for the organs at risk with the exception of the lung. The DVH of the lung for each patient was not significantly different among the various optimized plans. Different results were observed for the spinal cord, esophagus, and trachea DVHs in which it can be observed that the segment size had an influence in the dose. The location of the tumor relative to the heart for the sample patient makes it difficult to draw any conclusive results. Similar results can be observed for the rest of the patients. Figure 3 shows the DVHs of all eight plans for patient 1 for: (1) the PTV, (2) the lung, (3) spinal cord, (4) the esophagus, (5) the trachea, and (6) the heart.

### A.2 ${\text{Pinnacle}}^{3}$ and Monte Carlo simulations comparison

#### A.2.1 Patient dose calculation comparison

All the Monte Carlo dose calculations had a maximum dose uncertainty of less than 1%. The average minimum, maximum, and mean doses to the PTV for all of the patients were extracted from both ${\text{Pinnacle}}^{3}$ and Monte Carlo. The maximum and mean doses to the organs at risk were also reported (Table 6). ${\text{Pinnacle}}^{3}$‐calculated PTV mean doses were in agreement to Monte Carlo‐calculated mean doses to within 5.6% for all plans. On average, the mean dose difference between Monte Carlo and ${\text{Pinnacle}}^{3}$ for all 88 plans was 1.38%.

**Table 6:** Percent differences of mean dose values between ${\text{Pinnacle}}^{3}$ and MC calculation as a function of minimum segment size for the target and specific organs at risk.

		*PTV*		*Lung*		*Spinal Cord*	
*Plan*	*Min Ave*	*Max Ave*	*Mean Ave*	*Max Ave*	*Mean Ave*	*Max Ave*	*Mean Ave*
$0.10\;{\text{cm}}^{2}$	2.1±6.2	2.9±2.6	1.3±2.1	6.0±3	2.3±3.1	0.7±23.5	15.9±24.4
$0.25\;{\text{cm}}^{2}$	−0.3±8.9	1.0±3.6	−0.5±5.5	3.9±4.3	−0.1±7.2	12.4±25.3	18.2±30
$1\;{\text{cm}}^{2}$	4.0±6.2	3.7±2.1	2.7±2.9	6.4±3.4	3.5±3.1	11.0±25.5	16.4±30.6
$2\;{\text{cm}}^{2}$	−0.1±10.4	2.6±1.9	0.4±2.4	7.1±2.9	1.9±3.8	10.6±37.9	32.4±41
$3\;{\text{cm}}^{2}$	2.4±7.7	3.5±0.7	1.6±2.8	5.7±1.6	1.8±3.1	2.1±28	18.8±25.4
$4\;{\text{cm}}^{2}$	1.3±7.3	3.1±1.7	1.4±2.9	5.0±2.7	2.0±3.8	4.4±21.9	20.2±23.9
$5\;{\text{cm}}^{2}$	2.9±8.1	3.6±0.8	2.0±2.8	5.4±1.7	2.1±3.7	9.8±26.8	20.9±24.4
$6\;{\text{cm}}^{2}$	3.8±8.7	2.9±1.5	2.1±3.5	4.8±2.0	2.4±4.1	14.0±30.8	21.7±28.8
Mean	2.01±6.82	2.91±1.41	4.95±2.42	5.54±2.16	1.99±3.08	8.13±24.3	19.31±24.81

Higher discrepancies were observed for the minimum and maximum doses calculated. The largest discrepancy in maximum dose was 5.8% and was noted for one of the plans using a minimum segment size of $1.0\;{\text{cm}}^{2}$. For the minimum dose to the PTV, a maximum discrepancy between Monte Carlo and ${\text{Pinnacle}}^{3}$ was noted of 12.5% for a plan using a $6.0\;{\text{cm}}^{2}$ minimum segment size. Average differences for maximum and minimum doses to the PTV were 2.91 and 2.01%, respectively. Similar trends were observed for the organs at risk. It is important to mention that larger percentage discrepancies observed for some organs at risk are partly due to the fact that the absolute dose value is small. Hence, a relatively small absolute dose difference will lead to a relatively high percentage difference.

**Figure 4:**
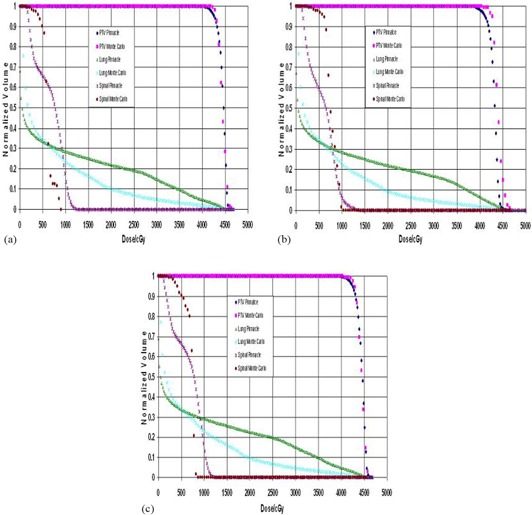
Dose volume histogram (DVH) comparison as a function of segment size between ${\text{Pinnacle}}^{3}$ and Monte Carlo calculations: (a) $0.25\;{\text{cm}}^{2}$, (b) $2\;{\text{cm}}^{2}$, and (c) $4\;{\text{cm}}^{2}$.

A DVH comparison for the PTV, spinal cord, and lung between ${\text{Pinnacle}}^{3}$ and Monte Carlo is presented in Fig. 4. The figure presents the comparison for three different segment sizes ($0.25\;{\text{cm}}^{2}$, $3\;{\text{cm}}^{2}$, and $4\;{\text{cm}}^{2}$). The ${\text{Pinnacle}}^{3}$ PTV coverage was in agreement to the Monte Carlo calculation for the $0.25\;{\text{cm}}^{2}$ and $4\;{\text{cm}}^{2}$ plans. Monte Carlo showed better coverage of the PTV for the $2\;{\text{cm}}^{2}$ plan. The spinal cord and the lung showed similar coverage for all plans. The Monte Carlo calculation gave a lower dose to the spinal cord compared to the ${\text{Pinnacle}}^{3}$ plan, and the lung received a larger dose with the Monte Carlo calculation as compared to the ${\text{Pinnacle}}^{3}$ plan.

**Figure 5:**
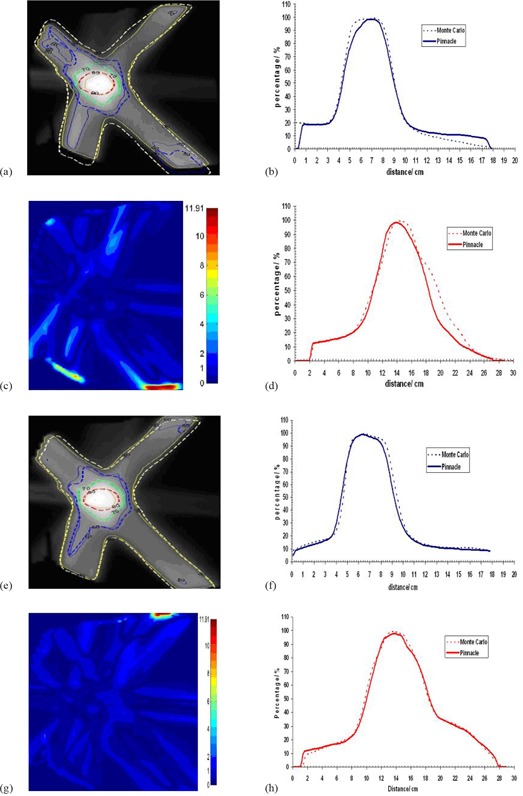
Dose comparison between MC and CCCS for patient 1 and for segment sizes $0.25\;{\text{cm}}^{2}$ and $4\;{\text{cm}}^{2}$, respectively: (a) and (e) isodose distributions; (b) and (f) horizontal profiles; (c) and (g) gamma images; (d) and (h) vertical profiles.

**Figure 6:**
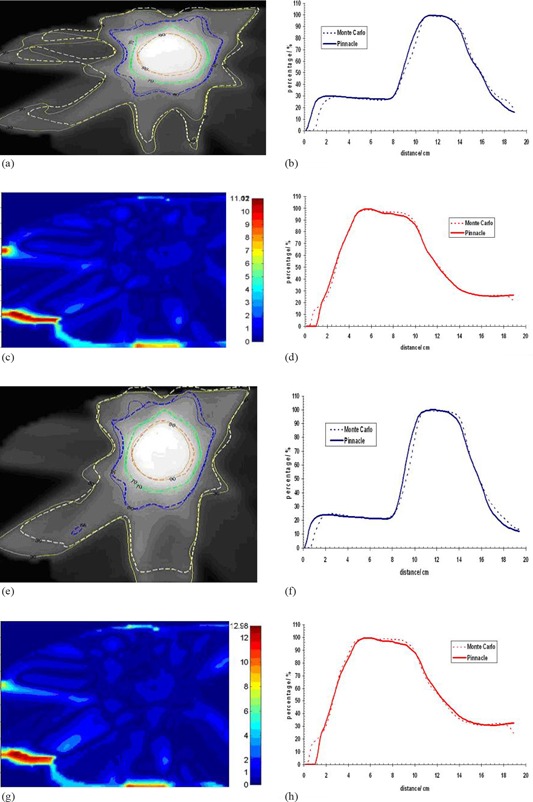
Dose comparison between MC and CCCS for patient 2 and for segment sizes $0.25\;{\text{cm}}^{2}$ and $4\;{\text{cm}}^{2}$, respectively: (a) and (e) isodose distributions; (b) and (f) horizontal profiles; (c) and (g) gamma images; (d) and (h) vertical profiles.

The ${\text{Pinnacle}}^{3}$ dose grid was exported and compared against the one calculated with Monte Carlo. The comparison between Monte Carlo and ${\text{Pinnacle}}^{3}$ planar isodose distributions were performed on the RITv5 software. An in‐house software was developed in MATLAB (v7.8 R2009a, The MathWorks, Natick, MA) to convert 3D dose distributions from Monte Carlo calculations into RITv5 readable format. Comparison was then possible on a slice‐by‐slice basis. The coronal, sagittal or transverse planes intersecting the beam isocenter were chosen for comparison. Representative isodose line comparisons are shown in Figs. 5 and 6 for different patients and minimum segment sizes. The corresponding gamma index, as well as horizontal and vertical profiles for these comparisons using a criterion of DTA 2 mm/percent dose difference 3%, is shown in Figs. 5 and 6. The profiles show a good agreement between ${\text{Pinnacle}}^{3}$ and Monte Carlo. The gamma index and the isodose lines support the result.

### A.3 Ion chamber and film measurements

Using both phantoms, measurements were acquired using a PTW 0.3 cc ionization chamber and radiographic EDR2 film. Point absolute dose measurements using the ionization chamber were made for all plans. Agreement between point dose measurements and ${\text{Pinnacle}}^{3}$ calculated doses were on average within 0.7% in both phantoms (see Table 7). Coronal planar doses at the level of the film were also exported. The dose to the center of the planar dose was compared between measurements and calculations. The agreement in this case was inferior to the point measurements. This is probably due to uncertainties in the film calibration curve and inaccuracy in determining the “center” of each step. Observing the absolute film doses at the center between the solid and lung phantom measurements and the respective TPS calculations, it is clear that better agreement is achieved in the case where the solid phantom was used. This is due to the difference in the phantoms composition and to the inhomogeneities embedded in the lung phantom (Solid Acrylic (Virtual Water, StandardImaging, Middleton, WI)) and two wood slabs lung tissue equivalent. This heterogeneous phantom gives the closest representation of a real clinical case in comparison to the solid water phantom in which case the difference in the results gives a significant representation of the actual plan delivered in a patient. These results are summarized in Table 7

**Table 7:** Percent differences of the average values between ${\text{Pinnacle}}^{3}$, ion chamber, and film.

	*Solid Phantom*		*Lung Phantom*	
*Plan*	*Ion Chamber vs. Pinnacle*	*Film vs. Pinnacle*	*Ion Chamber vs. Pinnacle*	*Film vs. Pinnacle*
$0.1\;{\text{cm}}^{2}$	0.7±5.6	0.7±7.8	−0.7±5.9	−5.7±9.8
$0.25\;{\text{cm}}^{2}$	−0.7±6.3	−2±7.7	−0.6±6	−2.6±16.6
$1.0\;{\text{cm}}^{2}$	−0.6±6.5	−5.5±10.3	0.6±8.4	−4.6±13.1
$2.0\;{\text{cm}}^{2}$	−0.5±7	−1.6±8.8	−1±7	−6.8±10.6
$3.0\;{\text{cm}}^{2}$	0.1±5.9	−2.4±5.8	−0.4±6.5	−5.4±10.3
$4.0\;{\text{cm}}^{2}$	0.1±6.1	−1.6±6.9	−0.3±6.3	−6.2±10
$5.0\;{\text{cm}}^{2}$	−0.1±7.6	−0.5±8	−0.4±6.7	−6.9±10
$6.0\;{\text{cm}}^{2}$	0.4±7.7	−1±7.8	−0.2±7.1	−6.7±10.2

**Figure 7:**
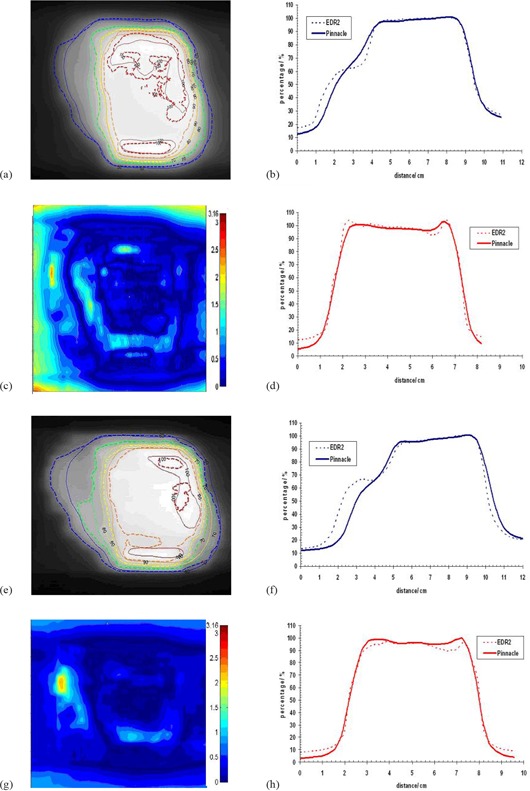
Dose comparison between film (EDR2) and CCCS for patient 1 and segment size $0.25\;{\text{cm}}^{2}$ for the solid water phantom (a–d) and the lung phantom (e–f): (a) and (e) isodose distributions; (b) and (f) horizontal profiles; (c) and (g) gamma images; (d) and (h) vertical profiles.

**Figure 8:**
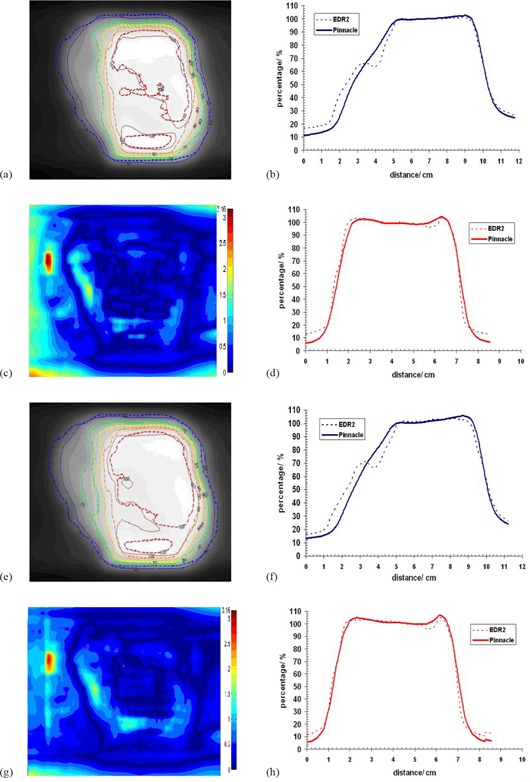
Dose comparison between film (EDR2) and CCCS for patient 1 and segment size $5\;{\text{cm}}^{2}$ for the solid water phantom (a–d) and the lung phantom (e–f): (a) and (e) isodose distributions; (b) and (f) horizontal profiles; (c) and (g) gamma images; (d) and (h) vertical profiles.

Planar doses exported from ${\text{Pinnacle}}^{3}$ were compared against their respective film measurements. The isodose distribution, gamma index using 2 mm DTA and 3% dose difference, and profiles were used for the evaluation of the TPS performance (Figs. 7 and 8). The figures show a good agreement between ${\text{Pinnacle}}^{3}$ and film measurements in the high‐dose region, in which case the result is confirmed with the isodose lines and gamma index. Small differences can be seen in the low‐dose regions which can be explained by the reasons stated above.

## IV. DISCUSSION

The average values of the percent difference between ${\text{Pinnacle}}^{3}$ and MC for the minimum, maximum, and mean doses characterizes the ability of ${\text{Pinnacle}}^{3}$ to accurately spare organs at risk while at the same time cover the PTV. The results showed that ${\text{Pinnacle}}^{3}$ is able to match the predictions made by the Monte Carlo simulations to within a few percent differences with the understanding that structures such as the spinal cord, trachea, and esophagus—typically small in volume—may in some circumstances be misleading. This is partly due to small differences in the conversion of the contours from ${\text{Pinnacle}}^{3}$ to Monte Carlo phantoms because of voxel size limitations which create potential discrepancies in the calculated volume. However, overall, high‐dose regions were accurately predicted by ${\text{Pinnacle}}^{3}$.

Superimpositions of the DVHs showed that ${\text{Pinnacle}}^{3}$ is able to obtain good coverage of the PTV independent of the segment size used. For the organs at risk, the TPS showed good agreement in the coverage of the total lung; as for the spinal cord, the location of the tumor influenced the dose statistics. ${\text{Pinnacle}}^{3}$ is able to deliver the prescribed dose to the PTV independent of the segment size used indicating that the planning system is able to model the difficulties associated with small fields.

The Monte Carlo codes EGSnrc\BEAMnrc and EGS4\MCSIM were used to calculate the particle transport to obtain the absorbed dose with the help of the ODMs by ${\text{Pinnacle}}^{3}$. These codes were used because they have shown that they provide the feasibility and flexibility to give accurate results. The number of histories used was chosen to provide a standard deviation of less than 2%. The improved Monte Carlo statistics lead to confirmation of good agreement among ${\text{Pinnacle}}^{3}$, MC and ion chamber measurements as shown in Table 7.

Agreement between point dose measurements and ${\text{Pinnacle}}^{3}$ calculated doses were, on average, within 0.7% in both phantom geometries. Better agreement was observed between ${\text{Pinnacle}}^{3}$ and the solid phantom as opposed to the inhomogeneous phantom. This is due probably to the difference in the phantom's composition and to the inhomogeneities embedded in the lung phantom. Using the inhomogeneous phantom, however, provides a closer representation of real patient anatomy.

## V. CONCLUSIONS

Agreement between ${\text{Pinnacle}}^{3}$ and Monte Carlo was within ±2% for the target mean dose, while higher discrepancies (up to 4%) were observed for the minimum and maximum doses. For the ipsilateral lung, a difference in the maximum dose of up to 7%, and 2% for the mean dose was noted. Small differences in the total coverage of the PTV were found for all cases as a function of segment size. Good agreement between ${\text{Pinnacle}}^{3}$ and MC using dose profiles, isodose distributions, and Gamma Index analysis verified the results—particularly in the high dose region.
